# Return to sport after ACL reconstruction, meniscus and cartilage surgeries in professional soccer players: a systematic review and meta-analysis

**DOI:** 10.1186/s43019-026-00304-w

**Published:** 2026-02-19

**Authors:** Riccardo D’Ambrosi, Jari Dahmen, Alessandro Carrozzo, Luca Maria Sconfienza, Christoph Kittl, Elmar Herbst, Christian Fink

**Affiliations:** 1 IRCCS Ospedale Galeazzi – Sant’Ambrogio, Milan, Italy; 2 Department of Biomedical Sciences for Health, University of Milan, Milan, Italy; 3 Department of Orthopedic Surgery and Sports Medicine, Amsterdam UMC location University of Amsterdam, Amsterdam, The Netherlands; 4 Musculoskeletal Health, Amsterdam Movement Sciences, Amsterdam, The Netherlands; 5 Academic Center for Evidence-based Sports medicine (ACES), Amsterdam, The Netherlands; 6 Amsterdam Collaboration on Health & Safety in Sports (ACHSS), IOC Research Center, Amsterdam, The Netherlands; 7 Dipartimento di Scienze della Vita, della Salute e delle Professioni Sanitarie, Università degli Studi “Link Campus University”, Città di Castello, Italy; 8 Department of Trauma, Hand and Reconstructive Surgery University of Muenster, Muenster, Germany; 9 Gelenkpunkt-Sports and Joint Surgery, FIFA Medical Centre of Excellence, Innsbruck, Austria; 10 Research Unit for Orthopaedic Sports Medicine and Injury Prevention (OSMI), Private University for Health Sciences Medical Informatics and Technology, Innsbruck, Austria

**Keywords:** Professional soccer players, Elite athletes, Meniscus injuries, Anterior cruciate ligament reconstruction, Cartilage procedures

## Abstract

**Background:**

The purpose of this systematic review and meta-analysis is to evaluate and compare the effects of anterior cruciate ligament reconstruction (ACLR), meniscal surgeries, and cartilage surgeries on return to sport (RTS) outcomes in professional soccer players.

**Materials and methods:**

The methodology followed the Preferred Reporting Items for Systematic Reviews and Meta-Analyses (PRISMA) guidelines. An electronic database search was performed to identify potentially relevant research articles. Four different outcome measures (age at surgery, return to sport, time to return to sport, level of return to sport) were extracted and meta-analyzed from all included studies and compared from three different groups (ACLR, cartilage surgeries, meniscus surgeries).

**Results:**

The pooled meta-analysis showed no difference in age at surgery among groups (*p* > 0.05). The overall pooled return-to-sport rate was 90% (95% CI 93.3–95.9), with no significant differences between ACL reconstruction, meniscus surgeries, and cartilage surgeries (*p* > 0.05) Patients treated for ACLR reported a longer time (*p* < 0.05) to return to sport (258.05 days; 95% CI 230.48–288.93) compared with meniscus (41.11 days; 95% CI 30.22–55.93) and cartilage surgeries (135.0 days; 95% CI 130.54–139.61). Furthermore, the pooled meta-analysis showed that athletes who underwent meniscus surgeries had a higher (*p* < 0.05) percentage of return to sport (100%: 95% CI 86.0–100.0) compared with ACLR (80.0%; 95% CI 67.5–90.3) and cartilage treatment (94.5%; 64.2–100.0).

**Conclusions:**

For professional soccer players, ACL reconstruction, meniscus surgeries, and cartilage surgeries demonstrated a favorable RTP rate of around 90%. Nevertheless, the analysis of the level of RTS and the time to RTS was constrained by limited evidence, precluding a more objective conclusion.

**Level of evidence:**

Meta-analysis of studies of Level IV.

Study Registration: PROSPERO Registry (CRD420251074362).

**Supplementary Information:**

The online version contains supplementary material available at 10.1186/s43019-026-00304-w.

## Introduction

Knee injuries represent one of the most common and debilitating conditions in professional soccer, frequently leading to prolonged absence from competition and, in the most severe cases, premature career termination [1–3]. Injuries to the anterior cruciate ligament (ACL), meniscus, and articular cartilage are particularly impactful due to their prevalence and the biomechanical demands of elite soccer [1–3].

ACL ruptures frequently require surgical reconstruction, with reported return-to-sport (RTS) rates of approximately 81–83% in elite athletes [4, 5]. However, only 55–65% return to their preinjury level of competition, partly owing to persistent neuromuscular deficits and psychological barriers such as fear of reinjury [6, 7].

Meniscal injuries are also highly prevalent in soccer, and surgical management is often required to preserve joint function [8]. While partial meniscectomy typically permits return to sport (RTS) within 6–7 weeks and repairs require 4–5 months [9], concerns remain regarding long-term joint health and the risk of early osteoarthritis, particularly in weight-bearing athletes [10, 11].

Articular cartilage lesions represent another significant challenge in elite soccer players due to limited intrinsic healing capacity [12]. RTS outcomes following cartilage procedures, including microfracture, osteochondral autograft transplantation, and autologous chondrocyte implantation, are variable, with reported rates ranging from 20 to 92% and recovery extending beyond 1 year in some cases [13–15].

Although RTS following individual procedures has been reported, no study has directly compared ACL reconstruction, meniscal surgery, and cartilage procedures in professional soccer players. This evidence gap limits optimal clinical decision-making and athlete counseling in cases involving isolated or combined injuries.

Therefore, the purpose of this systematic review and meta-analysis is to compare RTS outcomes—including return rate, time to return, and competitive level achieved—following ACL reconstruction, meniscal surgery, and cartilage procedures in professional soccer players. This analysis aims to provide clinicians with sport-specific prognostic information to guide treatment planning and athlete expectations.

## Material and methods

A systematic search strategy was developed according to the Preferred Reporting Items for Systematic Reviews and Meta-Analyses (PRISMA) guidelines and is registered in the PROSPERO Registry (CRD420251074362) [16, 17]. The AMSTAR-2 checklist was used to confirm the quality of the systematic review [18]. The TITAN checklist was fulfilled to transparently report the use of artificial intelligence [19]. An electronic database search was performed to identify potentially relevant research articles that analyzed return to sport, time taken to return to sport, level of return to sport, in professional soccer players after ACLR or cartilage, or meniscus surgeries. The MEDLINE (PubMed), Embase (Elsevier), and Cochrane Library databases were searched on 8 June 2025, and repeated after 2 weeks. A comprehensive literature search was conducted using Boolean operators with predefined concept groupings. The search strategy was structured as: (“ACL reconstruction” OR “anterior cruciate ligament reconstruction” OR “ACL” OR “cartilage” OR “meniscus”) AND “professional” OR “elite” OR “competitive”) AND (“soccer” OR “football”). This format ensured accurate logical grouping of terms and optimized retrieval of studies involving elite soccer athletes undergoing knee surgery.

### Eligibility criteria

The literature selected for this study was based on the following criteria.

The PICO framework for this review was defined as follows: the population consisted of skeletally mature professional or elite soccer players; the interventions included anterior cruciate ligament reconstruction, meniscus surgeries (meniscectomy or meniscal repair), and cartilage surgeries (microfracture, mosaicplasty, Hyalograft C or AMIC); the comparators were the three surgical categories evaluated against one another; and the outcomes included return-to-sport rate, time to return to sport, level of return to sport, and age at surgery. These elements were used to guide study selection, data extraction, and analysis.

### Study design

Studies were selected using predefined eligibility criteria. Randomized controlled trials, controlled clinical trials, prospective and retrospective cohort studies, case–control studies, and case series reporting on return to sport outcomes after ACLR or meniscus or cartilage surgeries were included. Case reports and case series were excluded if they lacked data on return to sport, time to return, or level of return. To avoid duplication, where multiple publications analyzed the same cohort with identical outcomes, only the most comprehensive or recent study was included.

### Participants and interventions

Eligible studies focused on skeletally mature elite soccer players who underwent primary ACLR or cartilage treatment or meniscus treatment and were assessed for return to sport parameters, including time to return, and level of return. Concomitant procedures were not considered exclusionary, provided that ACLR or meniscus or cartilage surgeries were the primary procedure. In trials that included revision ACLR without clearly separable data, outcomes were analyzed assuming primary surgery. An elite or professional athlete was defined as one who participates in national- or international-level competitions in professional or amateur sports—including academy players aged 15 years or over [20]. Minimum follow-up included studies that analyzed athletes for at least one full season after the intervention.

### Type of outcome measures

Four different outcome measures were extracted and recorded:

- Age at surgery
- Return to play: played in at least one game surgery
- Time to return to play: the time between surgery and return to the first official game
- Level of return to play: the criterion for return to preinjury level was based on playing at the same or higher level (% calculated on patients who returned to sports)

Studies were divided as follows on the basis of surgery:

- ACL reconstruction
- Cartilage surgery (regardless which type of surgery was performed)
- Meniscus surgery (regardless if meniscectomy or suture)

### Data collection and analysis

#### Study selection

The retrieved articles were first screened by title. If deemed relevant, they were further screened by reading the abstract. After excluding studies that did not meet the eligibility criteria, the entire content of the remaining articles was assessed for eligibility. To minimize the risk of bias, the authors reviewed and discussed all the selected articles, references, and articles excluded from the study. In case of any disagreement between the reviewers, the senior investigator made the final decision. At the end of the process, further studies that might have been missed were searched manually by going through the reference lists of the included studies and relevant systematic reviews.

### Data collection process

The data were extracted from the selected articles by the first two authors using a computerized tool created with Microsoft Access (Version 2010, Microsoft Corp, Redmond Washington). Each article was validated again by the first author before analysis. For each study, data regarding the patients were extracted, return to sport, time to return, and level of postoperative activity.

### Level of evidence

The Oxford Levels of Evidence set by the Oxford Center for Evidence-Based Medicine were used to categorize the level of evidence [21].

### Evaluation of the quality of studies

The quality of the selected studies was evaluated using the Methodological Index for Nonrandomized Studies (MINORS) score. The checklist includes 12 items, of which the last 4 are specific to comparative studies. Each item was given a score of 0–2 points. The ideal score was set at 16 points for noncomparative studies and 24 for comparative studies [22]. Two reviewers independently assessed the methodological quality of all included studies using the MINORS criteria. Any discrepancies in scoring were resolved through discussion to reach consensus, and when agreement could not be achieved, a third senior reviewer acted as an adjudicator.

#### Statistical analysis

Metanalyses were conducted overall and by group on (i) mean age; (ii) frequency of patients who returned to sport, (iii) average time to return to sport, (iv) frequency of patients who returned to the preinjury level.

For continuous outcomes, i.e. age and time to return to sport, we performed a random-effect model on log transformed means using the restricted maximum-likelihood (REML) estimator for variance estimation. The pooled estimates were presented as pooled means with 95% confidence intervals (CI). The metanalysis included primary studies with available standard deviation (SD) or range, from which we estimated the SD [23]. Time to return to sport was available on the subset of patients who returned to sport. Categorical outcomes were analyzed with a random-effects model using the Der Simonian–Laird estimator for the variance. The raw proportions were stabilized using the Freeman–Tukey double arcsine transformation. The pooled estimates were presented as pooled proportions with corresponding 95% CI. Frequency of patients who returned to the preinjury level was not available in all primary studies.

Differences among groups were explored with mixed-effects meta-regression models with common between-study variance component across groups, using variance estimators and transformations previously described. For each outcome, we tested within and between group heterogeneity with a Cochran’s Q *χ*^2^ test. Meta-regressions were performed with “ACL reconstruction” as reference category, thus comparing meniscus surgeries versus ACL reconstruction, and cartilage surgeries versus ACL reconstruction. The reference category was selected due to the highest frequency of studies and patients in this group.

Between-study variations were assessed for each model with the Cochran’s Q *χ*^2^ test of heterogeneity and the Higgins *I*^2^ statistic. Statistical heterogeneity was defined as substantial if *I*^2^ > 50% [24]. To further explore heterogeneity, we conducted a subgroup analysis by publication year, exploring studies published before 2021 and studies published in 2021 or later in separate groups (Supplementary Material). Publication bias and small-study effect were assessed through the funnel plot and DOI plot. Funnel plot symmetry was tested with rank correlation test and the regression test while the LKF index was calculated with the DOI plot. A sensitivity analysis with Trim-and-fill method was performed and the fail-safe N was calculated using the Rosenthal approach.

Two tailed tests were performed. A *p*-value of < 0.05 was considered to indicate statistical significance. The analysis was carried out using R (version 4.3.0, R Foundation for Statistical Computing, Vienna, Austria. URL https://www.R-project.org/) specifically with meta (version 8.0.1) and metafor packages (version 4.2.0).

## Results

**Fig. 1:**
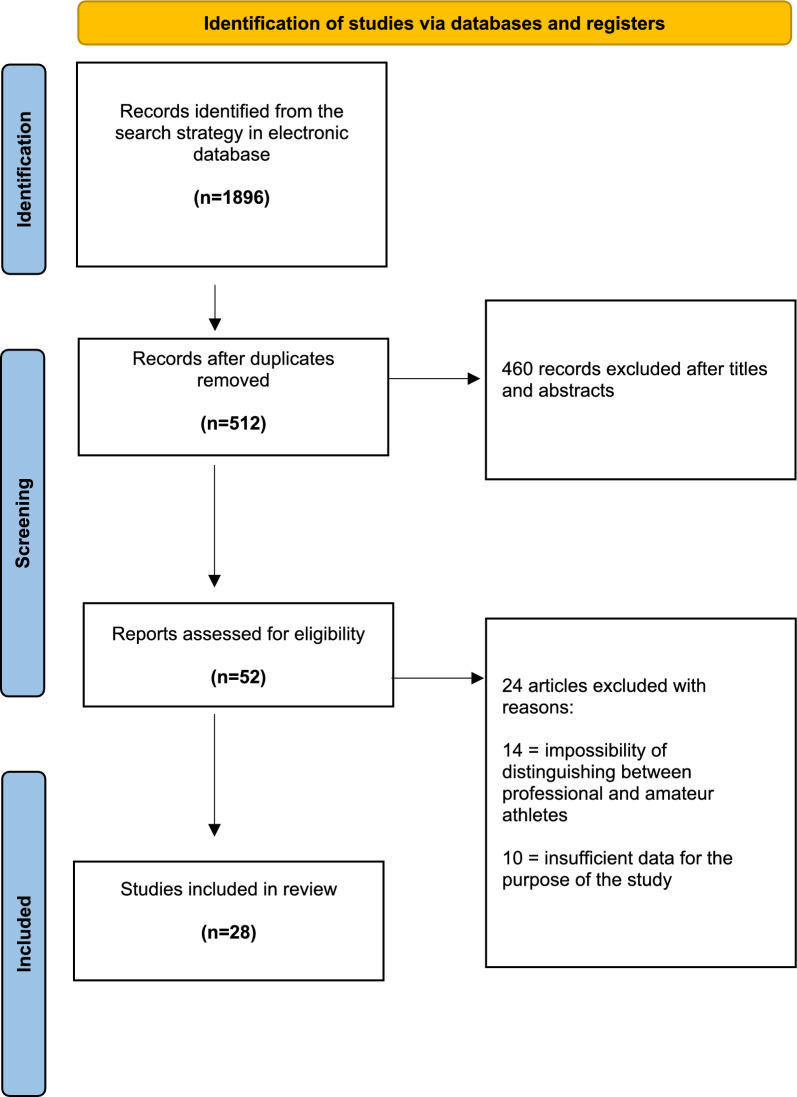
Preferred Reporting Items for Systematic Reviews and Meta-Analyses (PRISMA) flow chart indicating research article inclusion for final analysis

Initially, a thorough search of the three electronic databases yielded 1896 records. The titles and abstracts of 512 studies were reviewed after removing 1384 duplicates. After title and abstract screening, 1332 studies were removed, and 52 full-text articles were assessed for eligibility. Finally, the reviewers excluded 24 records after assessing the full texts, and 28 articles were included in the final analysis of this review [3, 5, 6, 20, 25–48]. The PRISMA diagram is shown in Fig. 1.

Of these, 20 analyzed ACLR, 4 meniscus surgery (but data from one study were considered separately), and 4 cartilage surgery (but data from one study were considered separately).

The inter-reviewer agreement for the MINORS assessment was very high (approximately 98%), with only minimal discrepancies between evaluators, all of which were resolved through consensus. Details of the studies are reported in Table 1.

**Table 1:** Analysis of each study included in the meta-analysis

Authors	MINORS	Level of evidence	Surgical procedure	Mean age (± SD)	% of return to sports	Time to return to sports (days (range) ± SD)	Same preinjury level
ACL reconstruction							
Erickson et al. 2013 [25]	14	III	ACLR	25.6 ± 3.98	78.9% (45/57)	n.a	95% (43/45)
Howard et al. 2016 [26]	14	IV	ACLR with autograft BPTB (52), autograft HT (13), autograft QT (1), allograft BPTB (1), allograft TA (1), allograft Achilles (3), allograft peroneal tendon (1), allograft HT (1), mixed allograft/autograft (2)	19.3 (17–22)	85% (66/78)	183 (117–996)	75% (50/66)
Walden et al. 2011 [5]	13	III	ACLR	24.3 ± 4.5	97.2% (69/71)	237.5 ± 76.1	95.6% (66/69)
Walden et al. 2016 [3]	15	III	ACLR	24.7 ± 4.5	97% (130/134)	201.5	64.5% (60/93)
Zaffagnini et al. 2014 [27]	15	IV	ACLR with HT	22.9 ± 5.4	71.4% (15/21)	186 ± 53	86.7% (13/15)
Arundale et al. 2018 [28]	15	III	ACLR with BPTB autograft (25), BPTP allograft (6), HT autograft (6)	n.a	74% (40/54)	n.a	90% (36/40)
Barth et al. 2019 [29]	15	IV	ACLR	26.1 ± 3.8	93.2% (164/176)	310.9 ± 14.9	100% (164/164)
Forsythe et al. 2021[30]	14	III	ACLR	24.9 ± 4.1	80% (41/51)	216 ± 109	100%(41/41)
Krutsch et al. 2020 [31]	12	IV	ACLR	24.8 ± 3.8	98.4% (62/63)	226.7 ± 93.5	62.9% (39/62)
Niedrer et al. 2018 [32]	13	III	ACLR	25.3 ± 4.2 (18–37)	98.2%(123/125)	209 ± 93	25.2% (31/123)
Abed et al. 2023 [33]	14	IV	ACLR	24.8 (22.5–28)	90% (27/30)	363 (327–429)	70.4% (19/27)
Balendra et al. 2022 [34]	14	IV	ACLR with HT (81), BPTB (150), allograft (1)	23.3 ± 4.4	96.1% (222/231)	315 ± 108	94.1% (209/222)
Bonanzinga et al. 2022 [35]	14	IV	ACLR with HT	25.3 ± 5.0	97% (27/28)	204 ± 108	47% (8/17)
Borque et al. 2024 [20]	15	III	ACLR with HT (25) or BPTB (57)	25.2 ± 4	98%(80/82)	327 ± 129	84% (67/80)
Farinelli et al. 2023 [36]	14	IV	ACLR with BPTB (17) or soft tissue QT (10)	23.15 ± 4.3 (18–34)	92.6% (25/27)	380	92% (23/25)
Ghali et al. 2025 [37]	14	IV	ACLR	25.3 ± 3.40	89.96% (20/23)	219	100% (20/20)
Jones et al. 2023 [38]	10	IV	ACLR with HT (81) or BPTB (154)	23.3 ± 4.3	95.7% (225/234)	318 ± 108	96.4% (217/225)
Mazza et al. 2022 [39]	12	IV	ACLR	25.4 ± 3.9 (18–37)	95% (174/183)	248 ± 136	47.7% (83/174)
Pinheiro et al. 2023 [6]	21	III	ACLR	24.1 ± 4.2	97% (194/200)	321 ± 117	35% (68/194)
Szymski et al. 2023 [40]	20	III	ACLR	24.7 ± 4.3 (18–32)	80% (96/120)	251.3 ± 79.0	50% (48/96)
Meniscus surgery							
Nawabi et al. 2014 (medial meniscus) [41]	20	III	Meniscectomy	22.4 ± 3.6	100% (48/48)	35 (21–42)	100% (48/48)
Nawabi et al. 2014 (lateral meniscus) [41]	20	III	Meniscectomy	23.7 ± 4.1	92.85% (39/42)	49 (35–126)	100% (39/39)
Heath et al. 2021 [42]	14	III	Medial meniscus repair	26.33 ± 4.35	83.77% (98/117)	165.9	100% (98/98)
Alvarez-Diaz et al. 2016 [44]	13	IV	All-inside meniscal repair	28 (18–37)	92% (13/14)	129	100% (13/13)
Cartilage surgery							
Pànics et al. 2012 [45]	13	IV	Mosaicplasty	25.3 ± 1.2	87% (53/61)	135 (105—183)	77.3% (41/53)
Mithoefer et al. 2012 [46]	14	IV	Microfracture	27 (18–32)	95% (20/21)	n.a	100% (20/20)
Bark et al. 2021 [47]	7	IV	AMIC	28	100% (1/1)	300	100% (1/1)
Kon et al. 2011 (microfracture) [48]	21	II	Microfracture	26.5 ± 4.5 (18–35)	80% (16/20)	240	15/16 (75%)
Kon et al. 2011 (Hyalograft C) [48]	21	II	Hyalograft C	23.7 ± 5.7 (16–37)	86% (18/21)	375	77.8% (14/18)

SD, standard deviation; AMIC, autologous matrix-induced chondrogenesis; ACLR, anterior cruciate ligament reconstruction; BPTB, Bone-patellar tendon-bone; HT, hamstring; QT, quadriceps tendon; TA, tibialis anterior

### Age

**Fig. 2:**
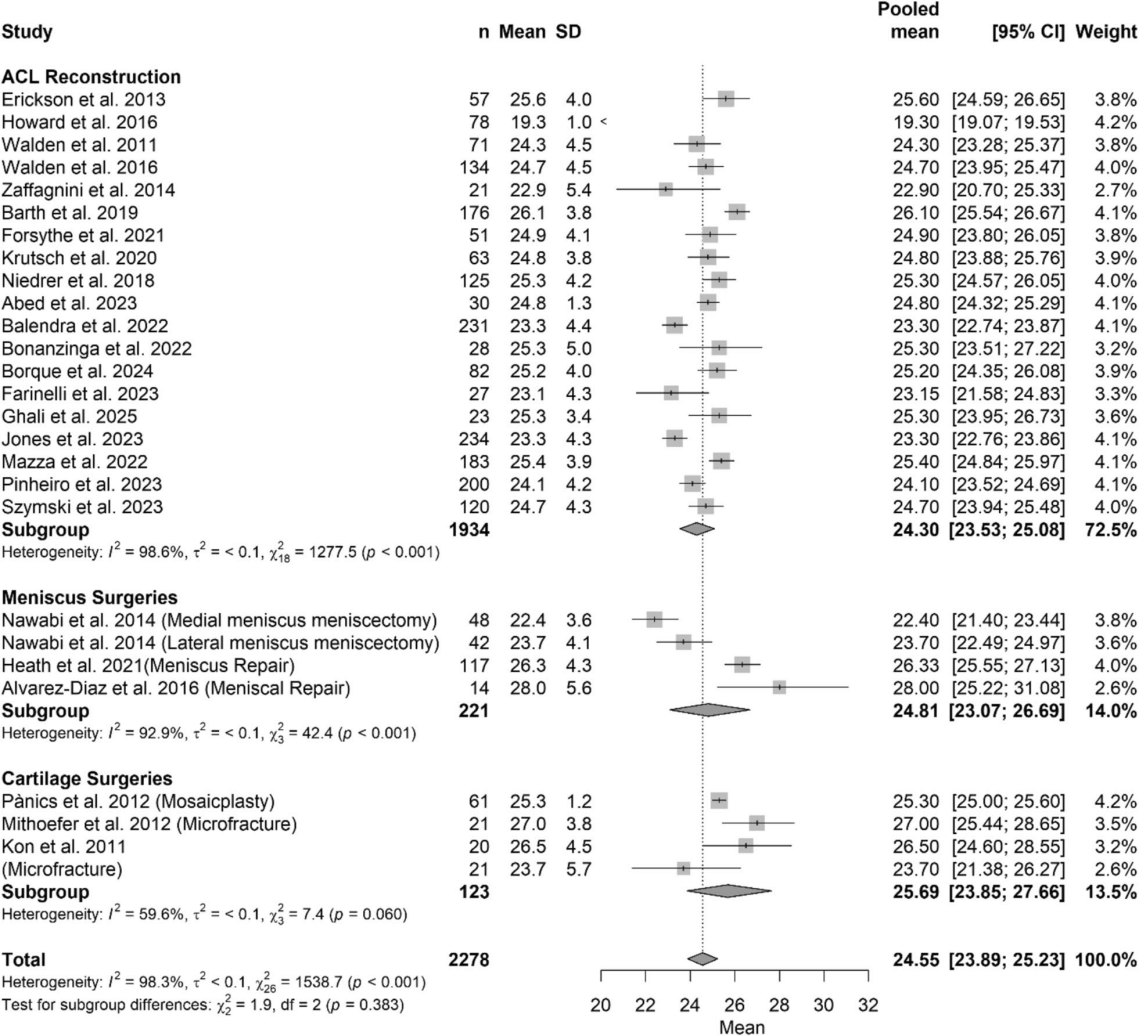
Forest plot of age among different treatments

The pooled meta-analysis showed no difference in term of age at surgery between the three different groups (*p* > 0.05). The Forest plot in Fig. 2 shows in details regarding age at surgery, while Table 2 shows beta coefficient.

**Table 2:** Meta-regression model comparing procedures (ACL reconstruction, meniscal surgery, and cartilage surgery) in relation to return to sport

Characteristics of the model		Values	
Tau^2^		0.0046 (SE = 0.0015)	
Tau		0.0678	
R^2^		0.00%	
Test for subgroup differences			
Within group		Q_24_ = 1327.31, *p*-value < 0.001*	
Between group		Q_2_ = 1.92, p-value = 0.383	
	N of studies	Beta coefficient [95%CI]^a^	*p*-value
Intercept (ACL reconstruction)	19	3.19 [3.16; 3.22]	Reference group
Meniscus surgeries	4	0.02 [−0.06; 0.10]	0.604
Cartilage surgeries	4	0.06 [−0.03; 0.14]	0.177

^a^beta coefficients are reported as log means. Beta coefficients are reported as log-means, with ACL reconstruction serving as the reference group. Positive values indicate a greater likelihood of return to sport relative to the reference, whereas negative values indicate a lower likelihood. 95% confidence intervals and corresponding *p*-values are provided for each comparison

*=statistical significant difference

### Return to sport

**Fig. 3:**
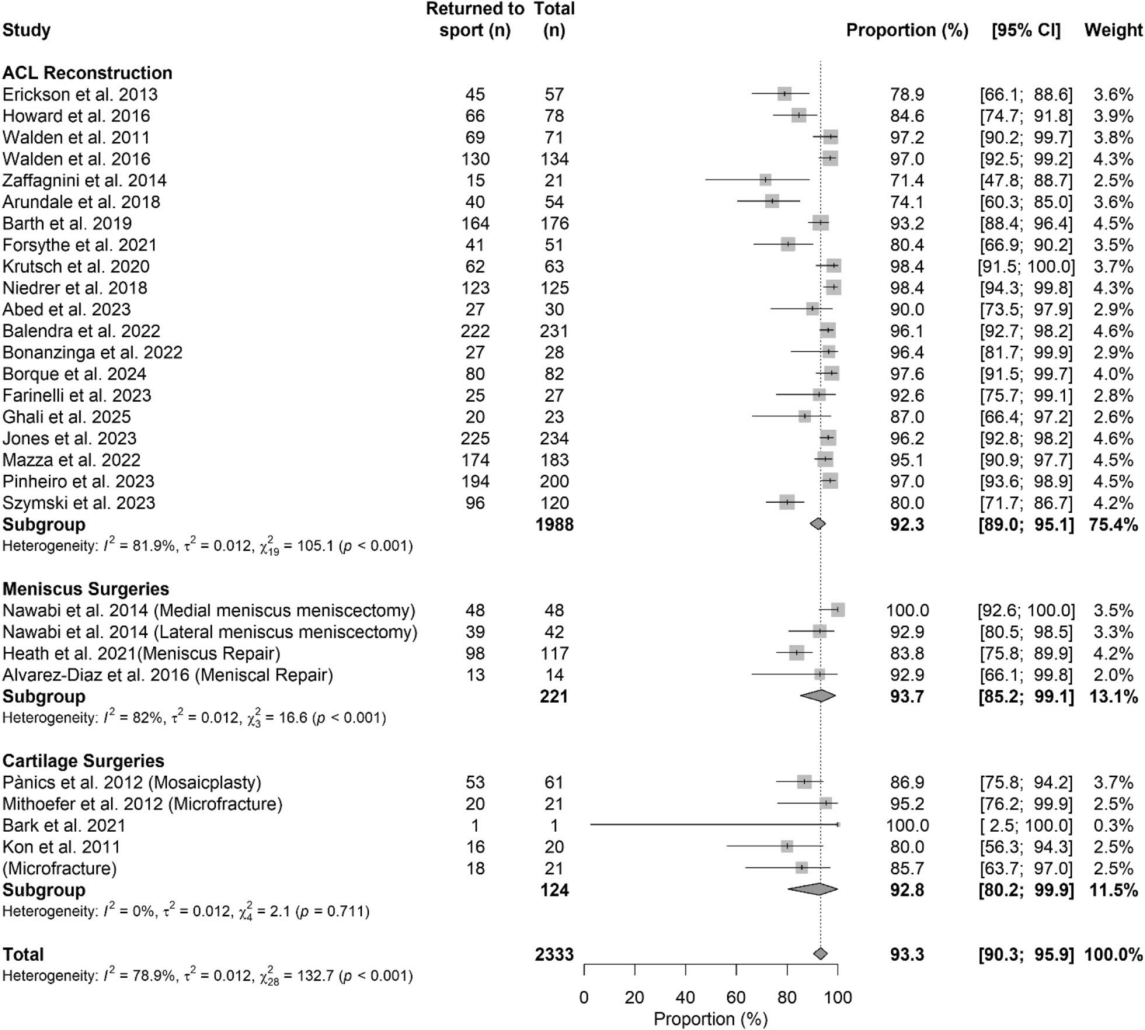
Forest plot of RTP among different treatments

The pooled meta-analysis showed no difference in term of percentage of return to sport between the three different groups (*p* > 0.05). The Forest plot in Fig. 3 shows details regarding return to sport, while Table 3 shows characteristics of the model and beta coefficient.

**Table 3:** Meta-regression model evaluating return to sport across surgical procedures

Characteristics of the model		Values	
tau^2^		0.012 (SE = 0.0049)	
R^2^		0.51%	
Test for subgroup differences			
Within group		Q_26_ = 123.87, *p*-value < 0.001*	
Between group		Q_2_ = 1.47, *p*-value = 0.480	
	N of studies	Beta coefficient [95%CI]^b^	*p*-value
Intercept (ACL reconstruction)	20	1.28 [1.22; 1.33]	Reference group
Meniscus surgeries	4	0.02 [−0.13; 0.16]	0.822
Cartilage surgeries	5	−0.09 [−0.24; 0.06]	0.251

^b^beta coefficients are reported as Freeman–Tukey double arcsine transformed proportion. Model characteristics, including between-study variance (tau^2^) and explained variance (*R*^2^), are reported together with tests for subgroup differences. Beta coefficients are presented using the Freeman–Tukey double arcsine transformed proportion, with ACL reconstruction as the reference category. Positive coefficients indicate a greater likelihood of return to sport relative to ACL reconstruction, whereas negative coefficients indicate a lower likelihood. 95% confidence intervals and *p*-values are provided for each estimate

*=statistical significant difference

### Time to return to sport

**Fig. 4:**
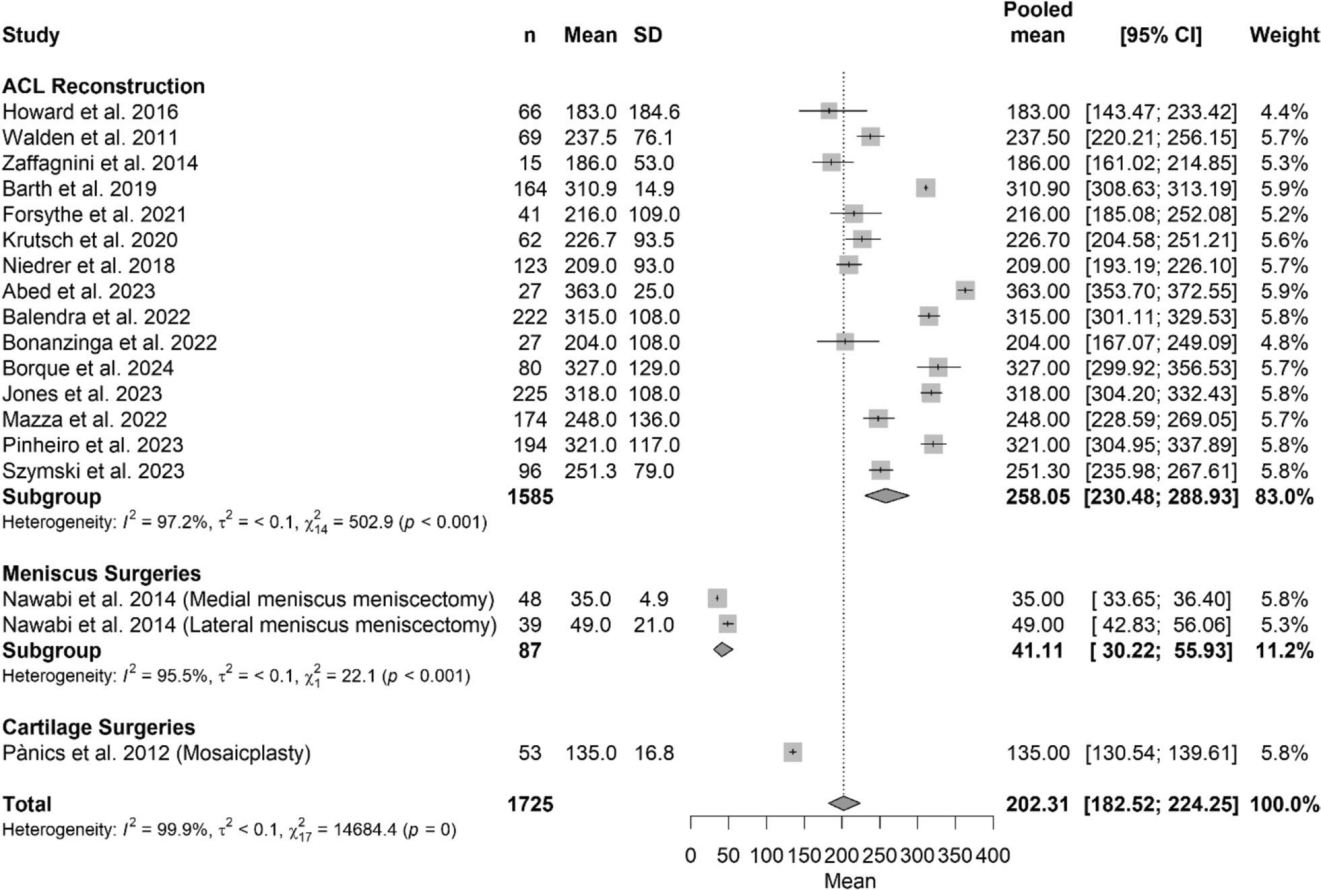
Forest plot of time to return to sport among different treatments

When analyzed by surgical group, professional soccer players who underwent ACL reconstruction demonstrated a pooled longer (*p* < 0.05) mean time to RTP of 258.05 days (95% CI 230.48–288.93), compared with meniscus surgeries (mean: 41.11 days [95% CI 30.22–55.93]) or cartilage surgeries (mean: 135.00 days [95%CI 130.54–139–61]) The Forest Plot in Fig. 4 shows details regarding time return to sport, while Table 3 shows characteristics of the model and Beta Coefficient.

### Analysis with meniscus and cartilage surgeries in the same group

**Fig. 5:**
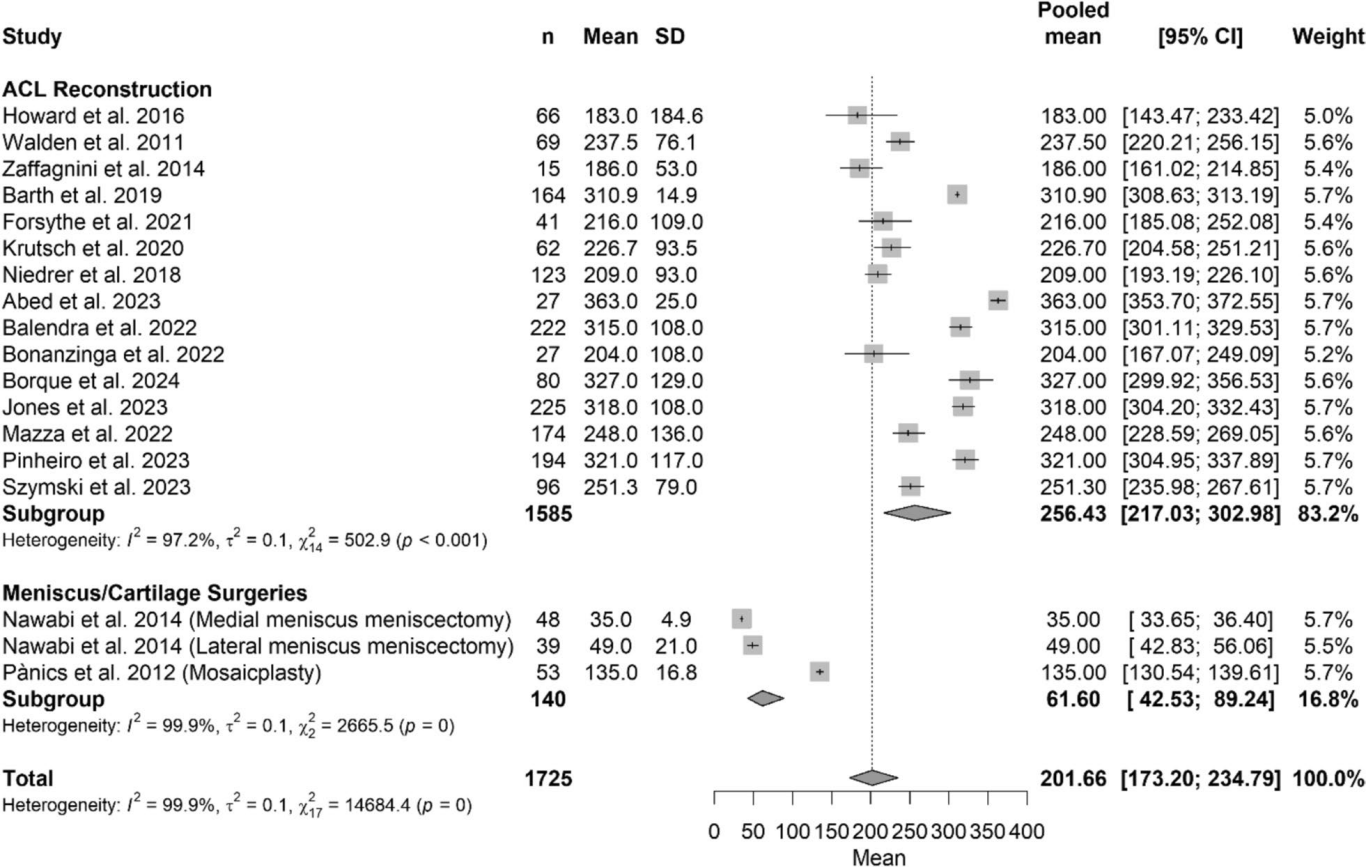
Forest plot of time to return to sport between ACL reconstruction versus meniscus/cartilage surgeries

When analyzed by surgical group, professional soccer players who underwent ACL reconstruction demonstrated a pooled longer (*p* < 0.05) mean time to RTP of 256.43 days (95% CI 217.03–302.98), compared with meniscus/cartilage surgeries group (mean: 61.60 days [95% CI 42.53–89.24]) or cartilage surgeries (mean: 135.00 days [95%CI 130.54–139–61]) The Forest plot in Fig. 5 shows details regarding time return to sport, while Table 4 shows characteristics of the model and Beta Coefficient.

**Table 4:** Meta-regression model evaluating time to return to sport across surgical procedures

Characteristics of the model		Values	
tau^2^		0.047 (SE = 0.018)	
tau		0.2165	
R^2^		88.22%	
Test for subgroup differences			
Within group		Q_15_ = 524.97, *p*-value < 0.001*	
Between group		Q_2_ = 124.26, *p*-value < 0.001*	
	*N* of studies	Beta coefficient [95%CI]^c^	*p*-value
Intercept (ACL reconstruction)	15	5.55 [5.44; 5.67]	Reference group
Meniscus surgeries	2	−1.84 [−2.16; –1.51]	< 0.001*
Cartilage surgeries	1	−0.65 [−1.09; –0.21]	0.004*

Model parameters, including between-study variance (tau^2^) and explained variance (*R*^2^), are reported along with subgroup heterogeneity statistics. Beta coefficients are expressed using the Freeman–Tukey double arcsine transformed metric, with ACL reconstruction designated as the reference category. Negative beta values indicate a shorter time to return to sport compared with ACL reconstruction, whereas positive values indicate a longer time. 95% confidence intervals and *p*-values are presented for each estimate

*=statistical significant difference

### Level of return to sport

**Fig. 6:**
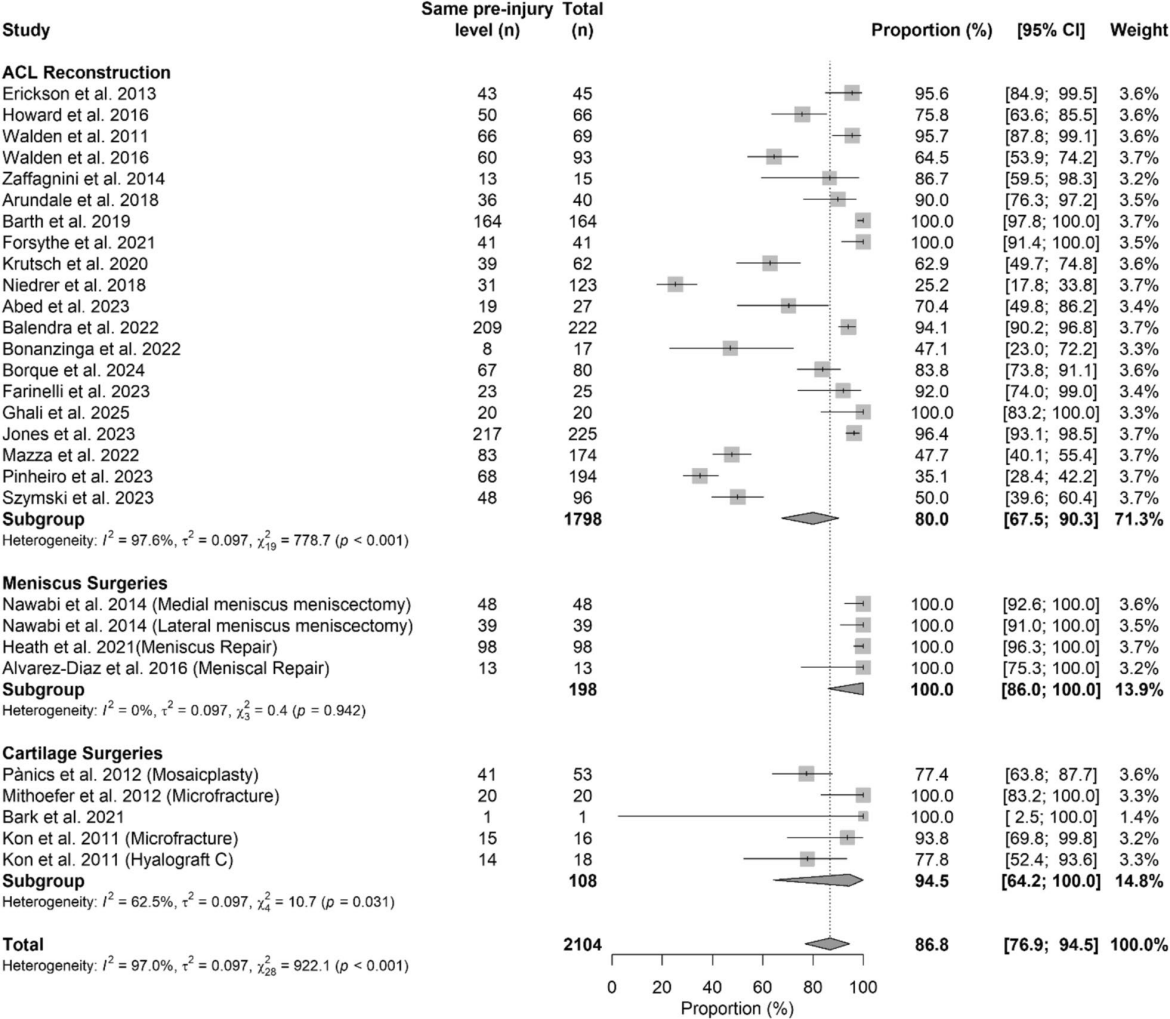
Forest plot of the level of RTP among different treatments

The pooled meta-analysis showed that athletes who underwent meniscus surgeries had a higher percentage of return to sport (100% [95% CI 86.0–100.0]) compared with ACL reconstruction (80.0% [95% CI 67.5–90.3]) and cartilage surgeries (94.5% [95% CI 64.2–100.0]) (*p* < 0.05). The Forest plot in Fig. 6 shows details regarding level of return to sport, while Table 5 shows characteristics of the model and Beta Coefficient. Table 6 shows characteristics of the model and Beta Coefficient.

**Table 5:** Meta-regression model evaluating return-to-sport level across surgical procedures

Characteristics of the model		Values	
tau^2^		0.097 (SE = 0.038)	
R^2^		9.25%	
Test for subgroup differences			
Within group		Q_26_ = 789.75, *p*-value < 0.001*	
Between group		Q_2_ = 4.68, *p*-value = 0.085	
	*N* of studies	Beta coefficient [95%CI]^e^	*p*-value
Intercept (ACL reconstruction)	20	1.10 [0.96; 1.24]	Reference group
Meniscus surgeries	4	0.39 [0.03; 0.74]	0.032*
Cartilage surgeries	5	0.11 [−0.23; 0.46]	0.529

Between-study variance (tau^2^), proportion of variance explained (*R*^2^), and heterogeneity statistics are reported. Beta coefficients are expressed as log means, with ACL reconstruction used as the reference category. Positive beta values indicate a higher level of return to sport relative to ACL reconstruction, whereas negative values indicate a lower level. 95% confidence intervals and *p*-values are provided for each estimate

*=statistical significant difference

**Table 6:** Meta-regression model assessing time to return to sport across surgical procedures

Characteristics of the model		Values	
tau^2^		0.1055 (SE = 0.0383)	
tau		0.3248	
R^2^		73.5%	
Test for subgroup differences			
Within group		Q_16_ = 3168.32, *p*-value < 0.001*	
Between group		Q_1_ = 47.30, *p*-value < 0.001*	
	*N* of studies	Beta coefficient [95%CI]^d^	*p*-value
Intercept (ACL reconstruction)	15	5.55 [5.38; 5.71]	Reference group
Meniscus/cartilage surgeries	3	−1.43 [−1.83; –1.02]	< 0.001*

^d^ beta coefficients are reported as log means. Model parameters, including between-study variance (tau^2^) and explained variance (*R*^2^), are reported together with heterogeneity statistics within and between subgroups. Beta coefficients are presented as log-means, with ACL reconstruction serving as the reference category. Negative beta values represent a shorter time to return to sport compared with ACL reconstruction, while positive values represent a longer time. Meniscal and cartilage surgery studies are combined into a single comparator category. 95% confidence intervals and *p*-values are reported for each estimate

*=statistical significant difference

## Discussion

This systematic review and meta-analysis examined return-to-sport outcomes in professional soccer players undergoing anterior cruciate ligament reconstruction, meniscal surgery, or cartilage procedures. Across all surgical categories, return-to-play rates were high, reinforcing that modern surgical and rehabilitation strategies permit elite footballers to resume competition following significant knee injury. However, the nature of the recovery trajectory differed substantially among procedures, particularly with respect to time to return and restoration of preinjury performance levels.

Athletes undergoing meniscal surgery demonstrated the most favorable short-term return profile, characterized by rapid return to competition and a high likelihood of reattaining preinjury competitive level. These findings align with prior evidence in high-demand athletes following partial meniscectomy, which consistently reports fast recovery timelines and efficient performance restoration [41]. The ability to load early and advance functional progression likely contributes to this accelerated return pathway. Nevertheless, such outcomes primarily reflect procedures that do not require biologic healing protection [49]. When repair is indicated, recovery duration increases markedly owing to the need to protect the repair site [42, 49]. Therefore, the favorable outcomes observed here should be interpreted as representative of specific meniscal indications amenable to expedited rehabilitation rather than all meniscal surgeries in elite players.

A similar pattern emerged for cartilage procedures. Professional athletes treated with osteochondral autograft transfer achieved a high rate of return to play with promising restoration of competitive performance. These findings corroborate prior literature suggesting that select osteochondral techniques can provide durable return to elite-level sport when applied to focal chondral defects in appropriately selected athletes [45, 46]. However, heterogeneity in biologic cartilage procedures warrants careful interpretation. Cell-based therapies and matrix-assisted techniques often require extended rehabilitation and biologic maturation timeframes [9, 50–52]. Consequently, the present results reflect specific high-yield indications in elite football rather than the broader spectrum of cartilage interventions.

In contrast, ACLR was supported by a larger evidence base and yielded more generalizable estimates of return-to-sport performance in professional footballers [8]. Most players returned to competitive activity; however, the time to return was substantially longer than for meniscal or osteochondral procedures, and a lower proportion regained their preinjury competitive level. These findings are consistent with large-scale studies demonstrating that only approximately two-thirds of elite players resume preinjury competitive status following ACL reconstruction due to persistent neuromuscular asymmetry, graft maturation considerations, psychological barriers, and constraints imposed by professional match-play demands [5, 27, 40, 53–58]. Contemporary evidence emphasizes that return to elite football after ACL reconstruction is not dictated solely by time, but by recovery of limb symmetry, movement quality, confidence, and progressive exposure to match-specific workloads [53–58].

Taken collectively, these results delineate distinct rehabilitation trajectories in elite football. Meniscal and osteochondral surgeries demonstrated accelerated short-term recovery profiles in selected contexts, whereas ACL reconstruction required longer rehabilitation and more stringent readiness criteria to support durable return-to-sport outcomes [41, 59–63]. These divergent profiles highlight the necessity of individualized treatment planning on the basis of injury pattern, tissue status, career timing, and long-term joint health objectives. Importantly, return-to-sport success should not be equated to full athletic recovery; return to performance requires restoration of physical capacity, psychological readiness, and sport-specific performance indices, particularly in the context of elite soccer competition [64, 65].

From a clinical perspective, the present findings offer practical benchmarks to support decision-making in the management of high-performance football athletes. Understanding differential recovery trajectories allows clinicians to provide accurate prognostic counseling, align expectations with competitive calendars, and guide return-to-performance strategies. Integrating strength benchmarks, neuromechanical assessments, psychological readiness measures such as the ACL-RSI, and controlled match-exposure progressions into clearance protocols may reduce reinjury risk and optimize long-term athletic success [66, 67]. In professional settings, collaborative communication between surgeons, physical therapists, performance scientists, coaching staff, and medical teams remains critical to ensure safe and sustainable recovery trajectories [68].

These results also reinforce the importance of individualized, procedure-specific management in elite football. When tissue quality and tear morphology permit, expedited management of isolated meniscal pathology may support rapid return without compromising athletic readiness, while cartilage restorative strategies may be prioritized in players with longer competitive horizons or structural preservation priorities. For ACL injuries, structured neuromuscular rehabilitation, objective clearance standards, and coordinated return-to-sport progression represent fundamental components of successful re-entry to elite play. Continued evolution toward personalized load management, data-driven recovery monitoring, and psychological readiness evaluation will likely further refine return-to-sport success in elite soccer populations.

Finally, the clinical relevance of these findings underscores the ability to tailor surgical strategy and rehabilitation to athlete profile, competition demands, and long-term joint preservation goals. Incorporating expected recovery timelines and performance trajectories into preoperative planning can optimize shared decision-making, enhance athlete confidence, and align stakeholder expectations across medical and coaching staff. In modern professional football, successful return encompasses not only participation but restoration of competitive performance and career longevity. Future research incorporating standardized return-to-sport definitions, performance-based metrics, and prospective methodology is essential to refine prognostic precision across surgical categories and deepen understanding of optimal recovery strategies in elite football athletes.

This study has several important limitations. First, substantial heterogeneity exists across the included studies regarding surgical techniques and perioperative strategies. For ACL reconstruction, variables such as graft type, fixation method, concomitant procedures including anterolateral ligament reconstruction or lateral extra-articular tenodesis, and rehabilitation protocols were not consistently reported and therefore could not be controlled for. Similarly, the meniscal and cartilage cohorts did not allow stratification by procedure type. Meniscectomy and meniscal repair were not differentiated, nor were tear pattern, repair technique, or tissue quality reported. Within the cartilage subgroup, distinctions among microfracture, autologous chondrocyte implantation, and osteochondral grafting techniques were not possible. This lack of granular detail limits the ability to draw procedure-specific conclusions.

Second, definitions of return to sport, duration of follow-up, and performance metrics varied across studies, with inconsistent reporting of key variables such as minutes played, match participation continuity, or validated functional scores. Psychological readiness measures were rarely included, despite their known relevance in elite athlete recovery. Furthermore, most included studies lacked prospective design or appropriate comparison groups, limiting causal inference.

Third, while the population analyzed consisted exclusively of professional soccer players, variability in league level, geographic regions, and competitive demands may influence external validity across elite football contexts. The inclusion of studies with concomitant procedures may also introduce clinical and methodological heterogeneity.

A critical limitation concerns the *time to return to sport* analysis. Only one eligible study was available for the meniscus category and one for the cartilage category, each involving procedures with the most accelerated recovery profile (partial meniscectomy and osteochondral autograft transfer, respectively). These highly selective data introduce risk of distortion and should be interpreted as exploratory and procedure-specific rather than representative of all meniscal or cartilage surgeries. As such, these findings should not be treated as primary conclusions of the study.

Finally, publication bias may favor studies reporting favorable outcomes in high-level athletes, although quality assessment and sensitivity analyses were performed to mitigate this effect. Despite these limitations, this study synthesizes available evidence in a homogeneous population of professional soccer players and provides clinically relevant benchmarks to support surgical decision-making, patient counseling, and return-to-performance planning in elite football.

## Conclusions

Professional soccer players demonstrate a high rate of return to play after anterior cruciate ligament reconstruction, meniscal surgery, and cartilage procedures, with overall RTP rates approaching 90%. However, interpretation of return-to-performance level and time to RTP must be approached with caution. The available evidence for meniscal and cartilage procedures is limited and largely reflects accelerated-recovery techniques applied in selected cases, precluding definitive procedure-specific conclusions. While these results provide useful benchmarking for elite-level rehabilitation planning, further high-quality, sport-specific research with standardized performance metrics and adequate representation of diverse surgical techniques is necessary to refine prognostic accuracy and guide evidence-based decision-making in professional football.

## Supplementary Information

Supplementary Material 1.

## Data Availability

Raw data are available upon request to the corresponding author.
