# Supplementary material for: Return to sport after ACL reconstruction, meniscus and cartilage surgeries in professional soccer players: a systematic review and meta-analysis
Source: Knee Surg Relat Res. 2026 Feb 19;38:6. doi: 10.1186/s43019-026-00304-w (PMC12922245; doi:10.1186/s43019-026-00304-w)
Supplement: Supplementary file 1 — Supplementary Material 1. [file 43019_2026_304_MOESM1_ESM.docx]

# Age

## Subgroup: publication year <2021

Number of studies: 15

Number of analysed patients: 952


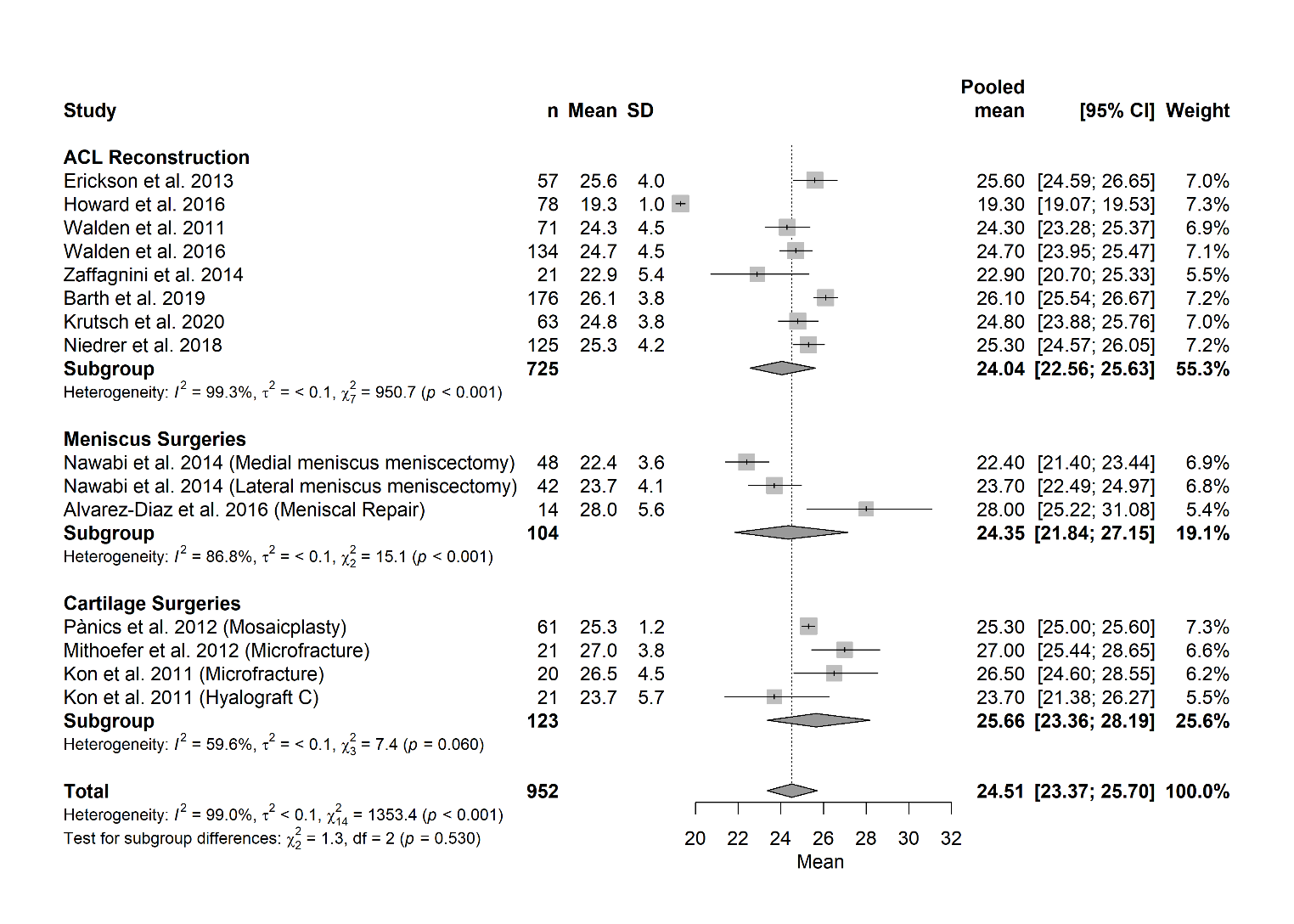


Estimates

|  | Beta coefficient [95%CI] ^1^ | p-value |
| --- | --- | --- |
| Intercept (ACL reconstruction) | 3.18 [3.12; 3.24] | Reference group |
| Meniscus Surgeries | 0.01 [-0.11; 0.14] | 0.845 |
| Cartilage Surgeries | 0.06 [-0.05; 0.18] | 0.263 |

Notes: ^1^ beta coefficients are reported as log means

## Subgroup: publication year >=2021 *

Number of studies: 12

Number of analysed patients: 1326


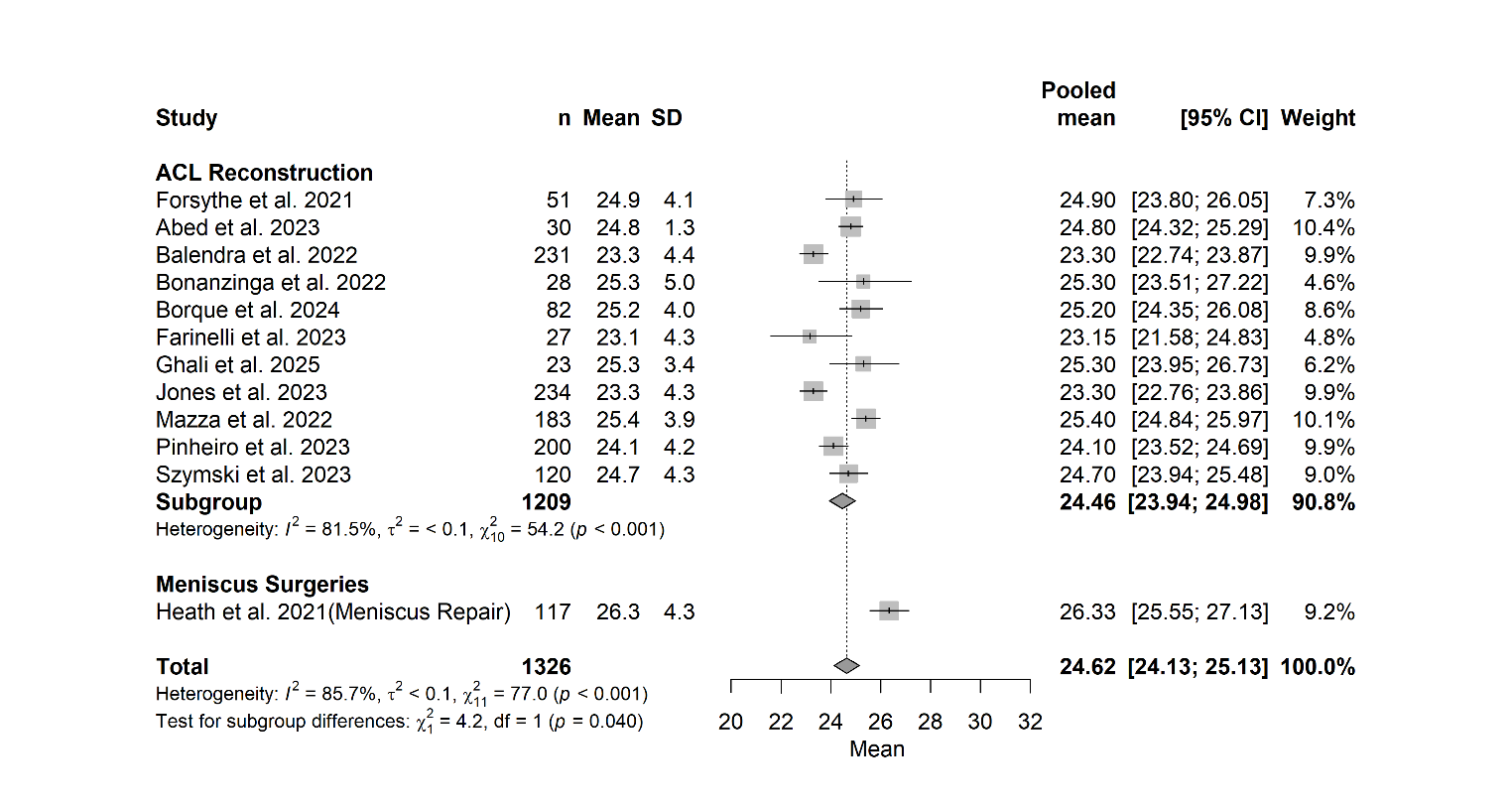


Estimates

|  | Beta coefficient [95%CI] ^1^ | p-value |
| --- | --- | --- |
| Intercept (ACL reconstruction) | 3.20 [3.18; 3.22] | Reference group |
| Meniscus Surgeries | 0.07 [0.01; 0.14] | 0.040* |

Notes: ^1^ beta coefficients are reported as log means

# % of return to sports

## Subgroup: publication year <2021

Number of studies: 16

Number of analysed patients: 1006

Number of patients returned to sport: 921


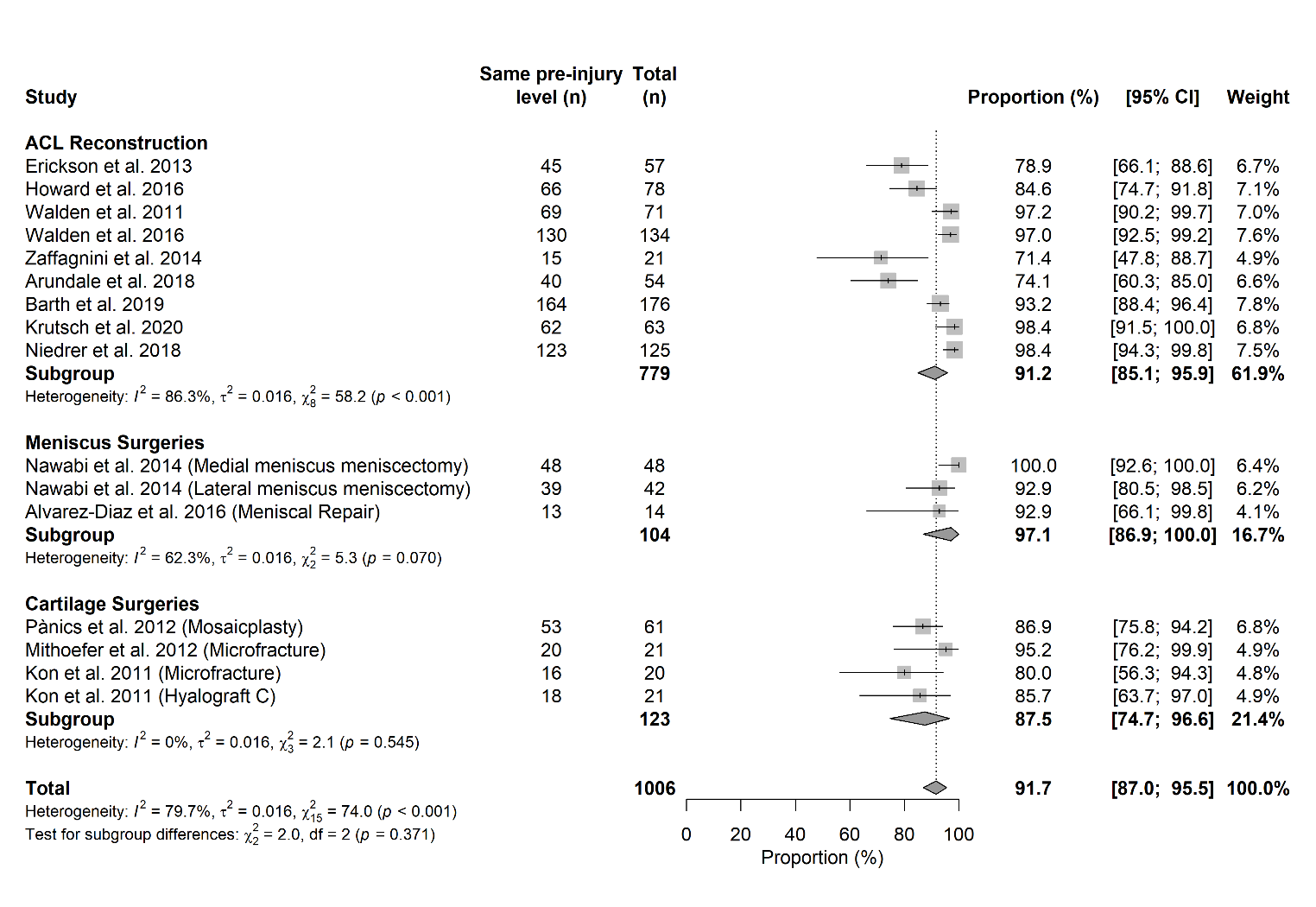


Estimates

|  | Beta coefficient [95%CI] ^1^ | p-value |
| --- | --- | --- |
| Intercept (ACL reconstruction) | 1.26 [1.17; 1.35] | Reference group |
| Meniscus Surgeries | 0.10 [-0.10; 0.30] | 0.324 |
| Cartilage Surgeries | -0.07 [-0.25; 0.11] | 0.455 |

Notes: ^1^ beta coefficients are reported as Freeman-Tukey double arcsine transformed proportion.

## Subgroup: publication year >=2021

Number of studies: 13

Number of analysed patients: 1327

Number of patients returned to sport: 1230


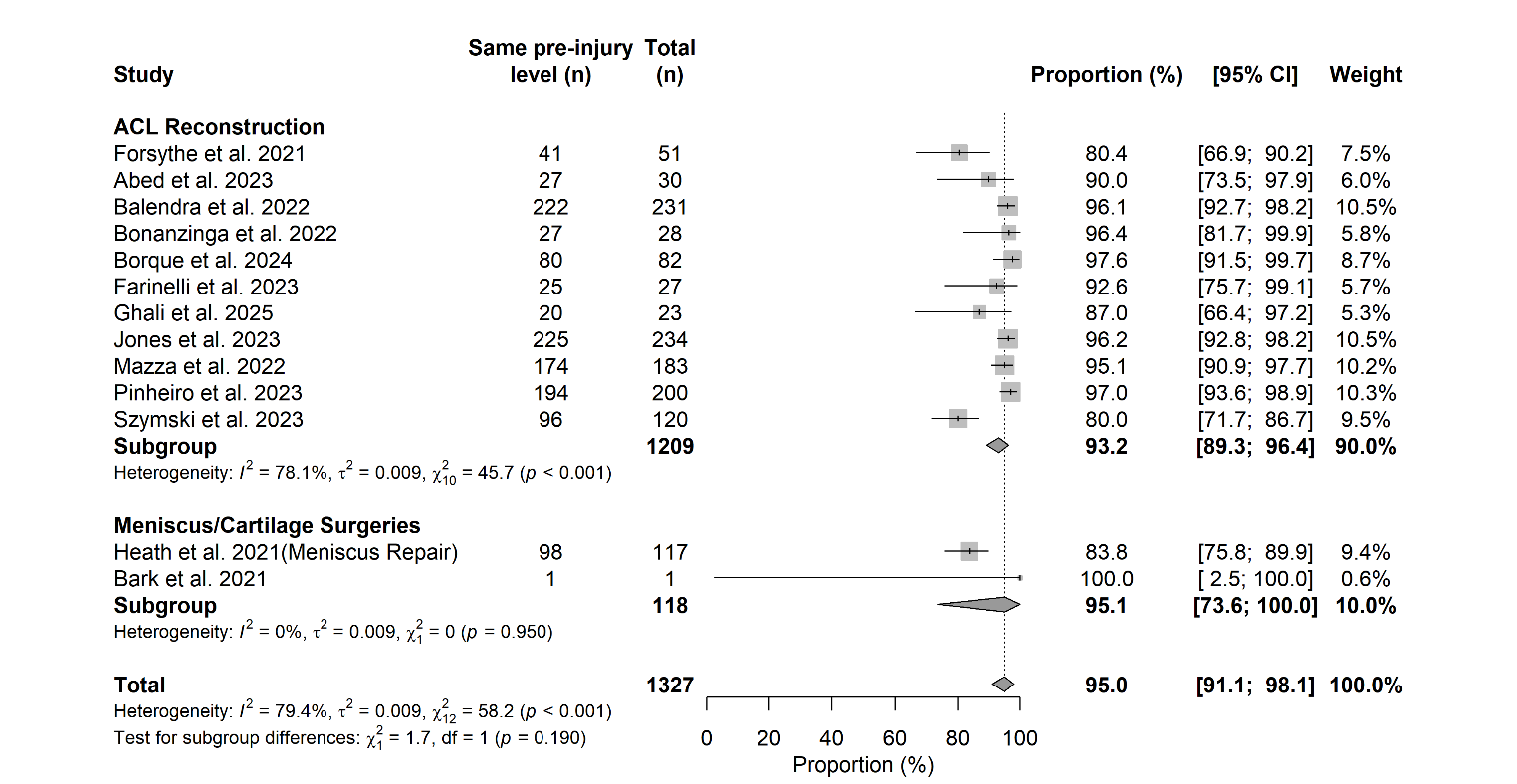


Estimates

|  | Beta coefficient [95%CI] ^1^ | p-value |
| --- | --- | --- |
| Intercept (ACL reconstruction) | 1.29 [1.23; 1.36] | Reference group |
| Meniscus /Cartilage Surgeries | -0.14 [-0.34; 0.07] | 0.184 |

Notes: ^1^ beta coefficients are reported as Freeman-Tukey double arcsine transformed proportion.

# Same pre-injury level*

## Subgroup: publication year <2021

Number of studies: 16

Number of analysed patients: 884

Number of patients returned to the same pre-injury level: 692


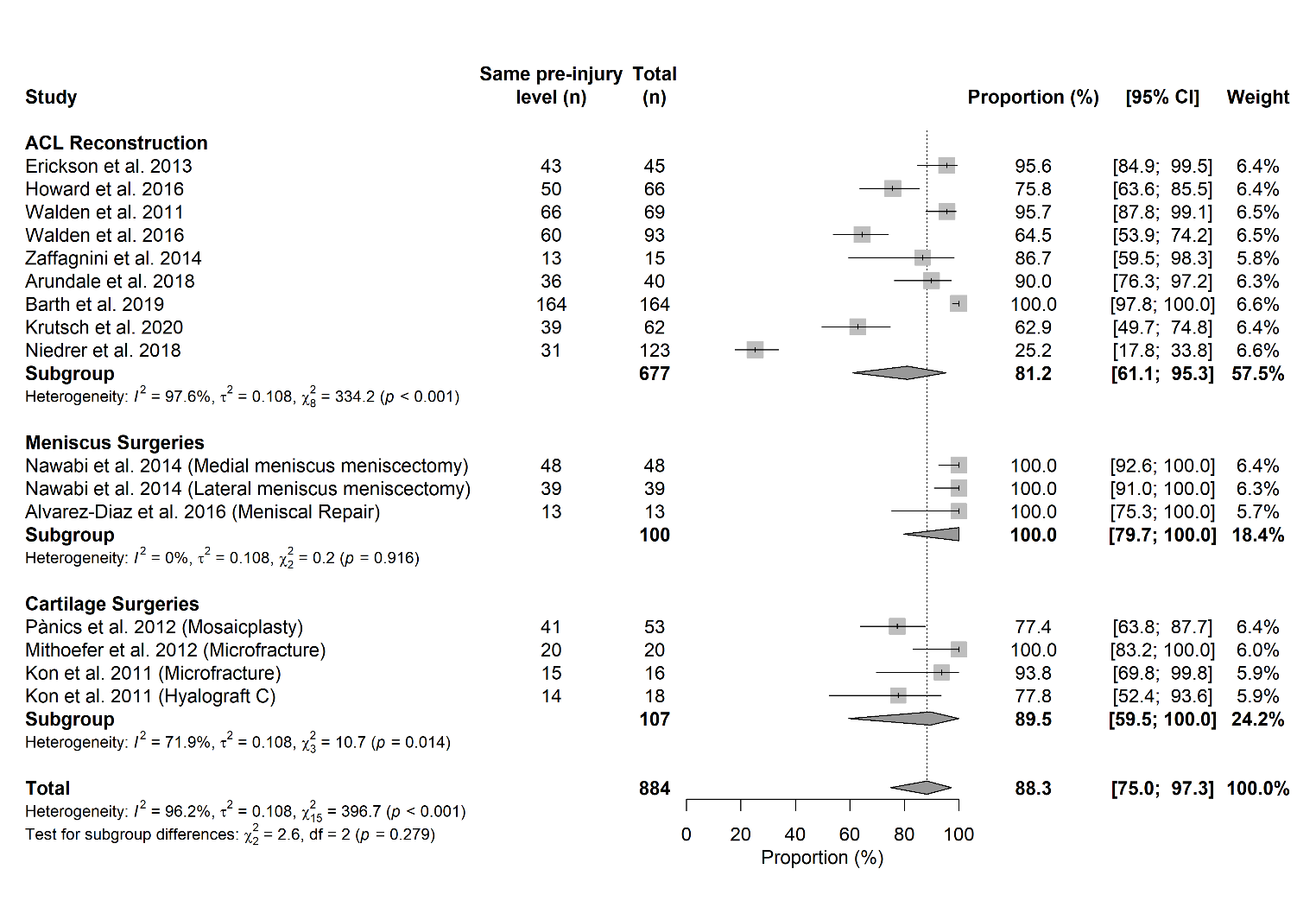


Estimates

|  | Beta coefficient [95%CI] ^1^ | p-value |
| --- | --- | --- |
| Intercept (ACL reconstruction) | 1.11 [0.89; 1.34] | Reference group |
| Meniscus Surgeries | 0.36 [-0.10; 0.82] | 0.123 |
| Cartilage Surgeries | 0.10 [-0.32; 0.52] | 0.637 |

Notes: ^1^ beta coefficients are reported as Freeman-Tukey double arcsine transformed proportion.

## Subgroup: publication year >=2021

Number of studies: 13

Number of analysed patients: 1220

Number of patients returned to the same pre-injury level: 902


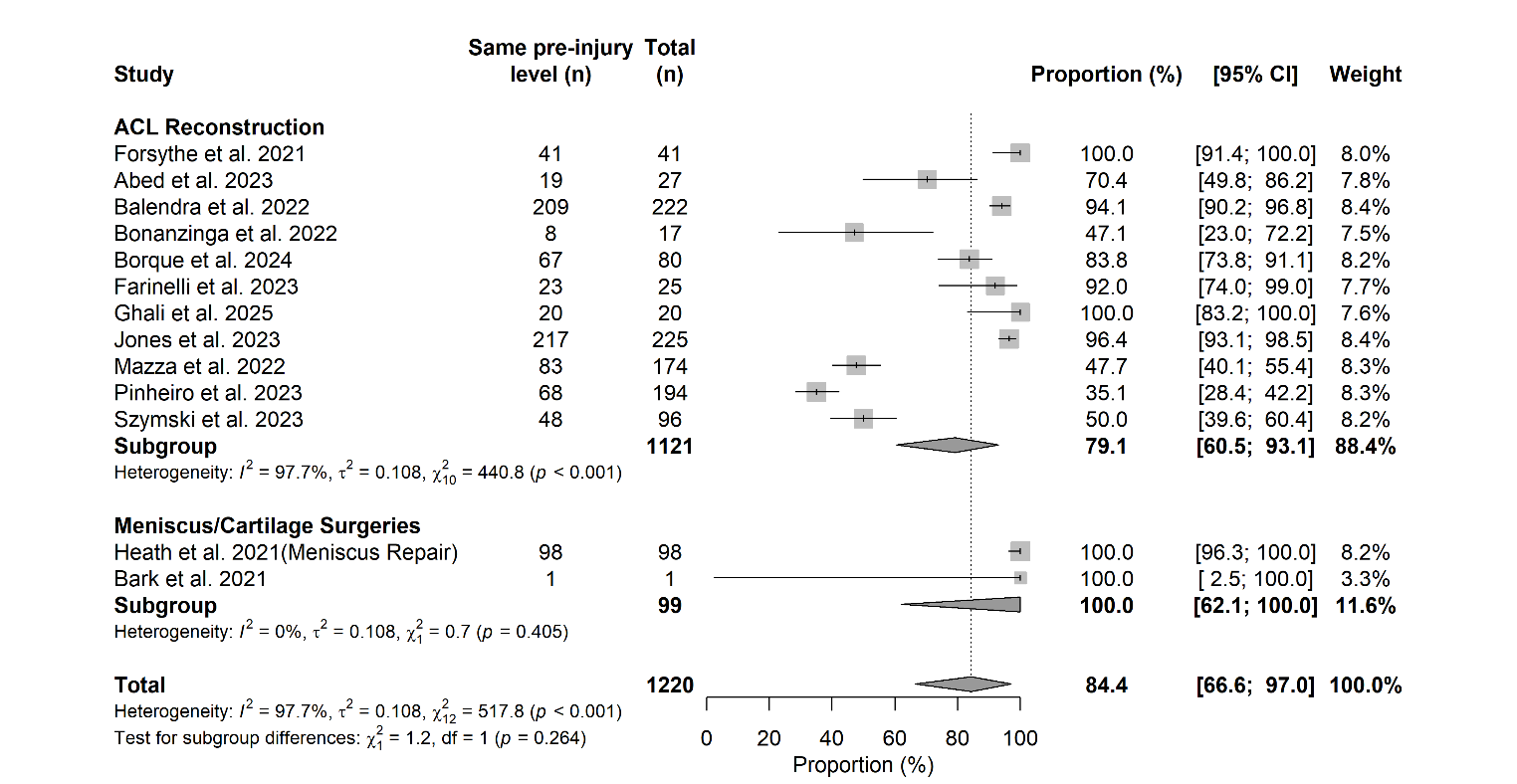


Estimates

|  | Beta coefficient [95%CI] ^1^ | p-value |
| --- | --- | --- |
| Intercept (ACL reconstruction) | 1.09 [0.89; 1.29] | Reference group |
| Meniscus / Cartilage Surgeries | 0.33 [-0.26; 0.93] | 0.264 |

Notes: ^1^ beta coefficients are reported as Freeman-Tukey double arcsine transformed proportion.

# Time to return to sport*

## Subgroup: publication year <2021*

Number of studies: 9

Number of analysed patients: 693


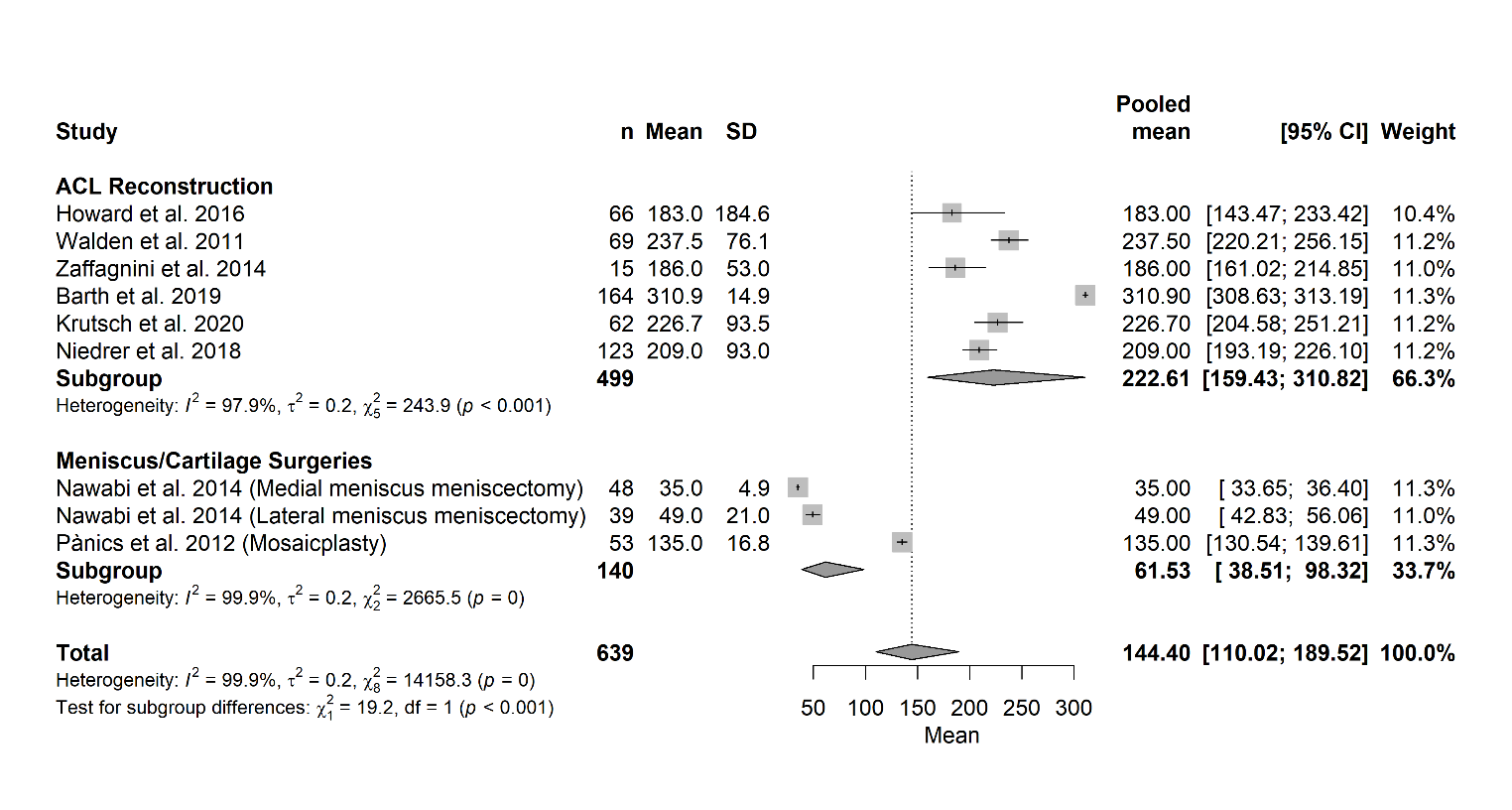


Estimates

|  | Beta coefficient [95%CI] ^1^ | p-value |
| --- | --- | --- |
| Intercept (ACL reconstruction) | 5.41 [5.07; 5.74] | Reference group |
| Meniscus/Cartilage Surgeries | -1.29 [-1.86; -0.71] | <0.001* |

Notes: ^1^ beta coefficients are reported as log means

## Subgroup: publication year >=2021

Number of studies: 9 -

Number of analysed patients: 1086


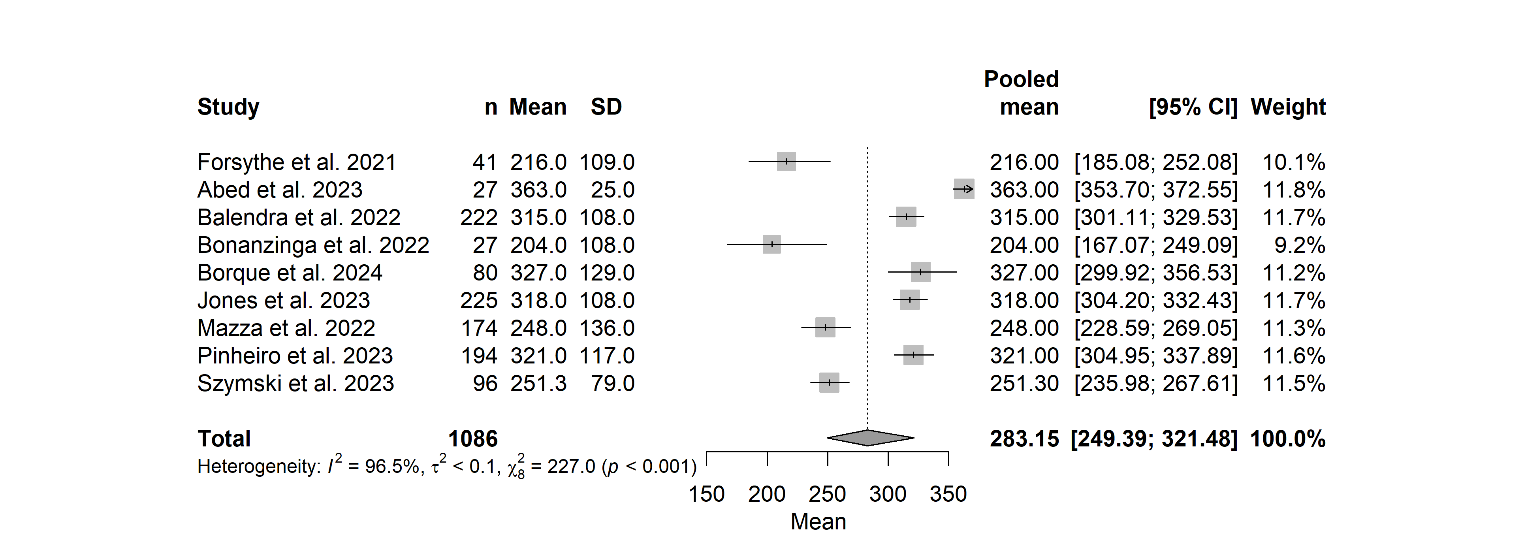


Notes: all studies in the figure are part of the group “ACL reconstruction”
